# Second monoclinic polymorph of 4-[(1*H*-benzimidazol-1-yl)meth­yl]benzoic acid

**DOI:** 10.1107/S1600536811045983

**Published:** 2011-11-05

**Authors:** Hai-Wei Kuai, Xiao-Chun Cheng

**Affiliations:** a Faculty of Life Science and Chemical Engineering, Huaiyin Institute of Technology, Huaian 223003, People’s Republic of China

## Abstract

Recently, we reported the first monoclinic [Kuai & Cheng (2011). *Acta Cryst.*, E**67**, o2787] and the ortho­rhom­bic polymorph [Kuai & Cheng (2011). *Acta Cryst.*, E**67**, o3014] of the title compound, C_15_H_12_N_2_O_2_. Another monoclinic polymorph was obtained accidentally by the hydro­thermal reaction of the title compound with manganese chloride in the presence of potassium hydroxide at 413 K. The asymmetric unit consists of four independent mol­ecules. In the crystal, O—H⋯N hydrogen bonds link the independent mol­ecules into four separate chains parallel to the *b* axis.

## Related literature

For the synthesis of 4-((1*H*-benzo[*d*]imidazol-1-yl)meth­yl)benzoic acid, see: Hua *et al.* (2010 ▶). For two other polymorphs of the title compound, see: Kuai & Cheng (2011*a* ▶,*b* ▶). For related structures, see Das & Bharadwaj (2009 ▶).
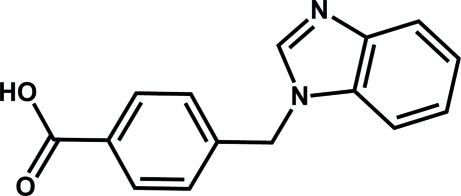


## Experimental

### 

#### Crystal data

C_15_H_12_N_2_O_2_
*M* *_r_* = 252.27
Monoclinic, 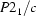

*a* = 16.704 (3) Å
*b* = 19.860 (3) Å
*c* = 15.343 (3) Å
β = 102.007 (3)°
*V* = 4978.5 (14) Å^3^
*Z* = 16
Mo *K*α radiation
μ = 0.09 mm^−1^
*T* = 293 K
0.20 × 0.20 × 0.18 mm

#### Data collection

Bruker SMART APEX CCD area-detector diffractometer
Absorption correction: multi-scan (*SADABS*; Sheldrick, 1996 ▶) *T*_min_ = 0.982, *T*_max_ = 0.984
25186 measured reflections
8754 independent reflections
3074 reflections with *I* > 2σ(*I*)
*R*_int_ = 0.069

#### Refinement

*R*[*F* ^2^ > 2σ(*F* ^2^)] = 0.055
*wR*(*F* ^2^) = 0.094
*S* = 0.88
8754 reflections
613 parameters
H-atom parameters constrained
Δρ_max_ = 0.53 e Å^−3^
Δρ_min_ = −0.48 e Å^−3^

### 

Data collection: *APEX2* (Bruker, 2008 ▶); cell refinement: *SAINT* (Bruker, 2008 ▶); data reduction: *SAINT*; program(s) used to solve structure: *SHELXS97* (Sheldrick, 2008 ▶); program(s) used to refine structure: *SHELXL97* (Sheldrick, 2008 ▶); molecular graphics: *DIAMOND* (Brandenburg, 2000 ▶); software used to prepare material for publication: *SHELXTL* (Sheldrick, 2008 ▶).

## Supplementary Material

Crystal structure: contains datablock(s) I, global. DOI: 10.1107/S1600536811045983/aa2025sup1.cif

Structure factors: contains datablock(s) I. DOI: 10.1107/S1600536811045983/aa2025Isup2.hkl

Supplementary material file. DOI: 10.1107/S1600536811045983/aa2025Isup3.cdx

Supplementary material file. DOI: 10.1107/S1600536811045983/aa2025Isup4.cml

Additional supplementary materials:  crystallographic information; 3D view; checkCIF report

## Figures and Tables

**Table 1:** Hydrogen-bond geometry (Å, °)

*D*—H⋯*A*	*D*—H	H⋯*A*	*D*⋯*A*	*D*—H⋯*A*
O1—H12⋯N12^i^	0.82	1.92	2.693 (4)	157
O3—H24⋯N111^ii^	0.82	1.85	2.613 (4)	154
O5—H36⋯N211^ii^	0.82	1.92	2.711 (4)	162
O7—H48⋯N311^iii^	0.82	1.84	2.628 (4)	160

Symmetry codes: (i) 
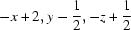
; (ii) 
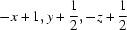
; (iii) 
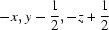
.

**Fig. 1.**
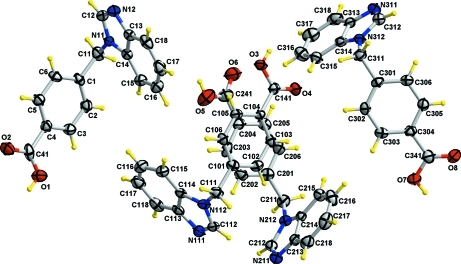
The crystal structure of the title compound showing 30% probability displacement ellipsoids.

**Fig. 2.**
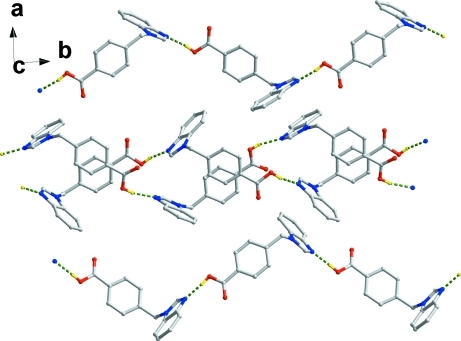
The packing diagram of the title compound. Hydrogen bonds are shown as dashed lines.

## supplementary crystallographic information

### Comment

The title compound, C_15_H_12_N_2_O_2_ (I), is usually regarded as an excellent candidate for building block in molecular self-assembly engineering due to its variable conformation and coordination modes (Das & Bharadwaj, 2009). During assembly of a coordination polymer, we accidentally obtained three polymorphs of (I), which can be proved by different unit-cell parameters and space groups. Here, we are introducing one of them. The single crystals of title compound were accidentally obtained by the hydrothermal reaction of the title compound with manganese chloride and 4,4'-bipyridine as an auxiliary ligand in the presence of potassium hydroxide at 413 K. In the crystal structure, the asymmetric unit consists of four independent molecules (Fig. 1). Hydrogen bonds link every kind of molecules in four separate chains parallel to *b* axis.(Fig 2).

### Experimental

Reaction mixture of MnCl_2_ (21.5 mg, 0.1 mmol), 4-((1*H*-benzo[*d*]imidazol-1-yl)methyl)benzoic acid (25.2 mg, 0.1 mmol), 4,4'-bipyridine (15.6 mg, 0.1 mmol) and KOH (5.61 mg, 0.1 mmol) in 10 ml H_2_O was sealed in a 16 ml Teflon-lined stainless steel container and heated to 413 K for 3 days. After cooling to the room temperature, colorless block crystals of the title compound were obtained.

### Refinement

All hydrogen atoms were located in geometrically idealized positions and constrained to ride on their parent atoms, with C—H = 0.93–0.97, O—H = 0.82 Å and *U*_iso_(H) = 1.2*U*_eq_(C, O).

### Crystal data

**Table:**

C_15_H_12_N_2_O_2_	*F*(000) = 2112
*M**_r_* = 252.27	*D*_x_ = 1.346 Mg m^−^^3^
Monoclinic, *P*2_1_/*c*	Mo *K*α radiation, λ = 0.71073 Å
Hall symbol: -P 2ybc	Cell parameters from 1589 reflections
*a* = 16.704 (3) Å	θ = 2.4–19.7°
*b* = 19.860 (3) Å	µ = 0.09 mm^−^^1^
*c* = 15.343 (3) Å	*T* = 293 K
β = 102.007 (3)°	Block, colorless
*V* = 4978.5 (14) Å^3^	0.20 × 0.20 × 0.18 mm
*Z* = 16	

### Data collection

**Table:**

Bruker SMART APEX CCD area-detector diffractometer	8754 independent reflections
Radiation source: fine-focus sealed tube	3074 reflections with *I* > 2σ(*I*)
graphite	*R*_int_ = 0.069
phi and ω scans	θ_max_ = 25.0°, θ_min_ = 1.6°
Absorption correction: multi-scan (*SADABS*; Sheldrick, 1996)	*h* = −19→19
*T*_min_ = 0.982, *T*_max_ = 0.984	*k* = −23→17
25186 measured reflections	*l* = −18→18

### Refinement

**Table:**

Refinement on *F*^2^	Primary atom site location: structure-invariant direct methods
Least-squares matrix: full	Secondary atom site location: difference Fourier map
*R*[*F*^2^ > 2σ(*F*^2^)] = 0.055	Hydrogen site location: inferred from neighbouring sites
*wR*(*F*^2^) = 0.094	H-atom parameters constrained
*S* = 0.88	*w* = 1/[σ^2^(*F*_o_^2^) + (0.0117*P*)^2^] where *P* = (*F*_o_^2^ + 2*F*_c_^2^)/3
8754 reflections	(Δ/σ)_max_ < 0.001
613 parameters	Δρ_max_ = 0.53 e Å^−^^3^
0 restraints	Δρ_min_ = −0.48 e Å^−^^3^

### Special details

**Table:**

Geometry. All s.u.'s (except the s.u. in the dihedral angle between two l.s. planes) are estimated using the full covariance matrix. The cell s.u.'s are taken into account individually in the estimation of s.u.'s in distances, angles and torsion angles; correlations between s.u.'s in cell parameters are only used when they are defined by crystal symmetry. An approximate (isotropic) treatment of cell s.u.'s is used for estimating s.u.'s involving l.s. planes.
Refinement. Refinement of *F*^2^ against ALL reflections. The weighted *R*-factor *wR* and goodness of fit *S* are based on *F*^2^, conventional *R*-factors *R* are based on *F*, with *F* set to zero for negative *F*^2^. The threshold expression of *F*^2^ > σ(*F*^2^) is used only for calculating *R*-factors(gt) *etc*. and is not relevant to the choice of reflections for refinement. *R*-factors based on *F*^2^ are statistically about twice as large as those based on *F*, and *R*- factors based on ALL data will be even larger.

### Fractional atomic coordinates and isotropic or equivalent isotropic displacement parameters (Å^2^)

**Table:**

	*x*	*y*	*z*	*U*_iso_*/*U*_eq_	
C1	0.9568 (2)	0.46606 (18)	0.3895 (2)	0.0422 (10)	
C2	0.9324 (2)	0.40495 (19)	0.4173 (2)	0.0582 (11)	
H1	0.8848	0.4019	0.4396	0.070*	
C5	1.0746 (2)	0.41250 (18)	0.3554 (2)	0.0550 (11)	
H3	1.1235	0.4158	0.3358	0.066*	
C6	1.0285 (2)	0.46969 (17)	0.3603 (2)	0.0497 (11)	
H4	1.0466	0.5111	0.3436	0.060*	
C341	−0.0552 (3)	0.3116 (2)	0.1259 (3)	0.067	
C11	0.9042 (2)	0.52842 (17)	0.3903 (2)	0.0550 (11)	
H6	0.8563	0.5167	0.4137	0.066*	
H5	0.9350	0.5620	0.4294	0.066*	
O6	0.51053 (18)	0.49299 (14)	0.3762 (2)	0.108	
C12	0.9020 (2)	0.61560 (18)	0.2701 (3)	0.0561 (11)	
H7	0.9355	0.6462	0.3064	0.067*	
C13	0.8267 (2)	0.56961 (17)	0.1573 (3)	0.0455 (10)	
C14	0.8284 (2)	0.52585 (18)	0.2284 (2)	0.0409 (10)	
C15	0.7865 (2)	0.46591 (18)	0.2207 (3)	0.0552 (11)	
H8	0.7880	0.4375	0.2692	0.066*	
C16	0.7424 (2)	0.4502 (2)	0.1374 (3)	0.0706 (14)	
H9	0.7135	0.4099	0.1291	0.085*	
C17	0.7397 (2)	0.4925 (2)	0.0656 (3)	0.0705 (14)	
H10	0.7088	0.4800	0.0104	0.085*	
C18	0.7811 (2)	0.5524 (2)	0.0729 (3)	0.0586 (12)	
H11	0.7790	0.5805	0.0240	0.070*	
C204	0.4889 (2)	0.37571 (19)	0.3588 (2)	0.057	
C101	0.5169 (2)	0.27039 (18)	0.0915 (2)	0.0426 (10)	
C102	0.4449 (2)	0.27237 (17)	0.1208 (2)	0.0468 (10)	
H13	0.4212	0.2323	0.1343	0.056*	
C103	0.4066 (2)	0.33315 (18)	0.1309 (2)	0.0441 (10)	
H14	0.3576	0.3336	0.1507	0.053*	
C104	0.4415 (2)	0.39316 (17)	0.1113 (2)	0.0354 (9)	
C105	0.5120 (2)	0.39092 (17)	0.0790 (2)	0.0469 (10)	
H15	0.5350	0.4308	0.0641	0.056*	
C106	0.5495 (2)	0.33045 (18)	0.0683 (2)	0.0503 (11)	
H16	0.5968	0.3299	0.0453	0.060*	
C111	0.5616 (2)	0.20400 (16)	0.0874 (2)	0.0528 (11)	
H17	0.5246	0.1716	0.0530	0.063*	
H18	0.6070	0.2109	0.0579	0.063*	
C112	0.5606 (2)	0.12699 (17)	0.2176 (3)	0.0549 (11)	
H19	0.5162	0.1014	0.1890	0.066*	
C113	0.6593 (2)	0.16490 (17)	0.3164 (3)	0.0456 (10)	
C114	0.6573 (2)	0.20312 (17)	0.2401 (2)	0.0416 (10)	
C115	0.7133 (2)	0.25315 (18)	0.2350 (3)	0.0612 (12)	
H20	0.7117	0.2779	0.1832	0.073*	
C116	0.7718 (3)	0.2642 (2)	0.3114 (3)	0.0810 (15)	
H21	0.8107	0.2976	0.3111	0.097*	
C117	0.7745 (3)	0.2275 (2)	0.3880 (3)	0.0771 (15)	
H22	0.8152	0.2366	0.4380	0.092*	
C241	0.5307 (3)	0.4410 (2)	0.3419 (3)	0.076	
C118	0.7184 (3)	0.1775 (2)	0.3922 (3)	0.0604 (12)	
H23	0.7202	0.1530	0.4443	0.072*	
C141	0.4015 (2)	0.45681 (18)	0.1305 (2)	0.0423 (10)	
C201	0.4000 (2)	0.25955 (19)	0.3761 (2)	0.048	
C202	0.4767 (2)	0.25568 (18)	0.3581 (2)	0.0516 (11)	
H25	0.4997	0.2136	0.3528	0.062*	
C4	1.0489 (2)	0.35122 (19)	0.3791 (2)	0.049	
C203	0.5214 (2)	0.31367 (18)	0.3476 (2)	0.0510 (11)	
H26	0.5726	0.3102	0.3331	0.061*	
C206	0.3681 (2)	0.32189 (19)	0.3862 (3)	0.0762 (14)	
H28	0.3162	0.3259	0.3986	0.091*	
C205	0.4133 (2)	0.3784 (2)	0.3777 (3)	0.076	
H27	0.3910	0.4203	0.3853	0.092*	
C211	0.3496 (2)	0.19836 (16)	0.3877 (2)	0.0479 (10)	
H30	0.3733	0.1764	0.4435	0.058*	
H29	0.2946	0.2124	0.3904	0.058*	
C212	0.3843 (2)	0.09039 (18)	0.3172 (3)	0.0504 (11)	
H31	0.4159	0.0726	0.3692	0.060*	
C213	0.3235 (2)	0.10285 (19)	0.1824 (3)	0.0453 (10)	
C214	0.3053 (2)	0.16015 (17)	0.2266 (2)	0.0376 (9)	
C215	0.2551 (2)	0.21126 (17)	0.1862 (3)	0.0507 (11)	
H32	0.2429	0.2486	0.2175	0.061*	
C216	0.2244 (2)	0.2034 (2)	0.0968 (3)	0.0645 (13)	
H33	0.1906	0.2366	0.0661	0.077*	
C41	1.0971 (3)	0.2881 (2)	0.3660 (3)	0.061	
C217	0.2424 (2)	0.1469 (2)	0.0504 (3)	0.0675 (13)	
H34	0.2201	0.1435	−0.0102	0.081*	
C218	0.2920 (2)	0.09597 (19)	0.0917 (3)	0.0607 (12)	
H35	0.3040	0.0586	0.0602	0.073*	
C301	0.1005 (2)	0.48270 (18)	0.1286 (2)	0.0437 (10)	
C302	0.1340 (2)	0.41913 (19)	0.1403 (2)	0.0533 (11)	
H37	0.1905	0.4136	0.1494	0.064*	
C303	0.0838 (2)	0.36324 (18)	0.1385 (2)	0.0546 (11)	
H38	0.1070	0.3206	0.1479	0.065*	
C3	0.9794 (2)	0.34748 (18)	0.4120 (2)	0.062	
H2	0.9630	0.3063	0.4311	0.074*	
C304	0.0000 (2)	0.37022 (18)	0.1230 (2)	0.0437 (10)	
C305	−0.0337 (2)	0.43360 (19)	0.1095 (2)	0.0506 (11)	
H39	−0.0902	0.4389	0.0981	0.061*	
C306	0.0163 (2)	0.48918 (18)	0.1128 (2)	0.0512 (11)	
H40	−0.0070	0.5318	0.1043	0.061*	
C311	0.1540 (2)	0.54473 (17)	0.1337 (2)	0.0542 (11)	
H41	0.2098	0.5311	0.1341	0.065*	
H42	0.1351	0.5723	0.0812	0.065*	
C312	0.0979 (2)	0.6337 (2)	0.2206 (3)	0.0668 (13)	
H43	0.0572	0.6477	0.1731	0.080*	
C313	0.1745 (3)	0.62597 (19)	0.3504 (3)	0.0530 (11)	
C314	0.2031 (2)	0.57861 (18)	0.2963 (3)	0.0457 (10)	
C315	0.2709 (2)	0.53916 (17)	0.3279 (3)	0.0575 (11)	
H44	0.2899	0.5078	0.2920	0.069*	
C316	0.3091 (3)	0.5489 (2)	0.4159 (3)	0.0704 (13)	
H45	0.3547	0.5230	0.4401	0.084*	
C317	0.2811 (3)	0.5962 (2)	0.4693 (3)	0.0742 (14)	
H46	0.3086	0.6014	0.5282	0.089*	
C318	0.2142 (3)	0.6352 (2)	0.4375 (3)	0.0693 (13)	
H47	0.1960	0.6670	0.4735	0.083*	
N11	0.87811 (17)	0.55688 (14)	0.30082 (19)	0.0438 (8)	
N112	0.59238 (18)	0.17763 (14)	0.17721 (19)	0.0444 (8)	
N211	0.37330 (18)	0.05956 (14)	0.2408 (2)	0.0538 (9)	
N212	0.34581 (17)	0.15062 (14)	0.31470 (19)	0.0407 (8)	
N311	0.1079 (2)	0.65961 (15)	0.3005 (3)	0.0693 (11)	
N312	0.15250 (19)	0.58474 (14)	0.2135 (2)	0.0490 (9)	
O1	1.06387 (15)	0.23355 (12)	0.38492 (16)	0.0820 (9)	
H12	1.0923	0.2014	0.3768	0.098*	
O2	1.16183 (16)	0.29344 (12)	0.34088 (17)	0.0769 (9)	
O3	0.44324 (13)	0.51106 (11)	0.11685 (14)	0.0546 (7)	
H24	0.4191	0.5448	0.1283	0.066*	
O4	0.33852 (16)	0.45866 (11)	0.15714 (17)	0.0621 (8)	
O5	0.58857 (17)	0.43358 (13)	0.30521 (17)	0.093	
H36	0.6093	0.4703	0.2995	0.112*	
N12	0.87382 (18)	0.62591 (14)	0.1846 (2)	0.055	
O7	−0.01721 (15)	0.25792 (12)	0.15838 (18)	0.0939 (11)	
H48	−0.0503	0.2274	0.1581	0.113*	
N111	0.5975 (2)	0.11677 (14)	0.3007 (2)	0.056	
O8	−0.12811 (17)	0.31467 (12)	0.0998 (2)	0.0977 (11)	

### Atomic displacement parameters (Å^2^)

**Table:**

	*U*^11^	*U*^22^	*U*^33^	*U*^12^	*U*^13^	*U*^23^
C1	0.040 (3)	0.048 (3)	0.036 (2)	0.009 (2)	0.002 (2)	−0.0038 (19)
C2	0.044 (3)	0.063 (3)	0.071 (3)	0.003 (2)	0.019 (2)	0.009 (2)
C5	0.054 (3)	0.053 (3)	0.060 (3)	0.007 (2)	0.015 (2)	0.003 (2)
C6	0.060 (3)	0.039 (3)	0.051 (3)	0.007 (2)	0.013 (2)	0.011 (2)
C341	0.071	0.058	0.067	−0.004	0.004	0.002
C11	0.055 (3)	0.061 (3)	0.045 (3)	0.010 (2)	0.000 (2)	−0.007 (2)
O6	0.104	0.062	0.169	−0.009	0.051	−0.001
C12	0.044 (3)	0.037 (3)	0.086 (3)	−0.002 (2)	0.009 (2)	−0.008 (2)
C13	0.043 (3)	0.039 (3)	0.054 (3)	0.007 (2)	0.008 (2)	0.002 (2)
C14	0.037 (2)	0.036 (2)	0.049 (3)	0.0044 (19)	0.007 (2)	0.000 (2)
C15	0.052 (3)	0.041 (3)	0.069 (3)	−0.002 (2)	0.006 (2)	0.006 (2)
C16	0.056 (3)	0.059 (3)	0.088 (4)	−0.005 (2)	−0.004 (3)	−0.013 (3)
C17	0.060 (3)	0.079 (4)	0.063 (3)	0.016 (3)	−0.009 (3)	−0.016 (3)
C18	0.060 (3)	0.065 (3)	0.050 (3)	0.020 (2)	0.012 (2)	0.011 (2)
C204	0.067	0.040	0.058	−0.004	0.000	0.005
C101	0.050 (3)	0.040 (3)	0.037 (2)	0.003 (2)	0.007 (2)	−0.0026 (19)
C102	0.052 (3)	0.040 (3)	0.049 (3)	−0.005 (2)	0.012 (2)	0.0025 (19)
C103	0.038 (2)	0.049 (3)	0.047 (2)	0.002 (2)	0.0141 (19)	0.000 (2)
C104	0.035 (2)	0.034 (2)	0.036 (2)	−0.0007 (19)	0.0045 (19)	−0.0059 (18)
C105	0.050 (3)	0.035 (2)	0.056 (3)	−0.003 (2)	0.013 (2)	−0.002 (2)
C106	0.045 (3)	0.048 (3)	0.063 (3)	0.001 (2)	0.022 (2)	−0.007 (2)
C111	0.061 (3)	0.044 (3)	0.052 (3)	0.004 (2)	0.009 (2)	−0.006 (2)
C112	0.048 (3)	0.030 (2)	0.090 (3)	−0.004 (2)	0.021 (2)	−0.003 (2)
C113	0.045 (3)	0.039 (3)	0.055 (3)	0.013 (2)	0.015 (2)	0.001 (2)
C114	0.044 (3)	0.028 (2)	0.054 (3)	0.003 (2)	0.013 (2)	−0.004 (2)
C115	0.056 (3)	0.045 (3)	0.079 (3)	−0.004 (2)	0.006 (3)	0.007 (2)
C116	0.067 (4)	0.054 (3)	0.111 (4)	−0.012 (3)	−0.007 (3)	−0.005 (3)
C117	0.075 (4)	0.057 (3)	0.086 (4)	0.014 (3)	−0.014 (3)	−0.020 (3)
C241	0.076	0.062	0.085	−0.005	0.008	−0.017
C118	0.069 (3)	0.058 (3)	0.052 (3)	0.025 (3)	0.009 (3)	0.001 (2)
C141	0.045 (3)	0.041 (3)	0.038 (2)	−0.002 (2)	0.003 (2)	−0.001 (2)
C201	0.059	0.048	0.037	0.000	0.011	−0.003
C202	0.059 (3)	0.042 (3)	0.056 (3)	0.004 (2)	0.018 (2)	−0.010 (2)
C4	0.040	0.051	0.053	0.000	0.002	−0.012
C203	0.046 (3)	0.067 (3)	0.044 (2)	−0.014 (2)	0.018 (2)	−0.011 (2)
C206	0.053 (3)	0.054 (3)	0.126 (4)	−0.006 (2)	0.029 (3)	−0.016 (3)
C205	0.060	0.050	0.115	0.009	0.011	−0.003
C211	0.053 (3)	0.051 (3)	0.043 (2)	−0.005 (2)	0.017 (2)	−0.006 (2)
C212	0.045 (3)	0.045 (3)	0.060 (3)	0.000 (2)	0.008 (2)	0.012 (2)
C213	0.041 (3)	0.045 (3)	0.050 (3)	−0.007 (2)	0.010 (2)	−0.009 (2)
C214	0.033 (2)	0.039 (2)	0.042 (2)	−0.0047 (19)	0.0089 (19)	0.003 (2)
C215	0.051 (3)	0.045 (3)	0.057 (3)	0.005 (2)	0.012 (2)	0.001 (2)
C216	0.057 (3)	0.079 (4)	0.054 (3)	0.013 (3)	0.006 (3)	0.014 (3)
C41	0.036	0.093	0.050	0.010	−0.003	−0.014
C217	0.063 (3)	0.096 (4)	0.043 (3)	−0.014 (3)	0.008 (2)	0.001 (3)
C218	0.057 (3)	0.067 (3)	0.061 (3)	−0.010 (2)	0.018 (2)	−0.020 (2)
C301	0.047 (3)	0.042 (3)	0.045 (2)	−0.008 (2)	0.016 (2)	0.0002 (19)
C302	0.042 (3)	0.050 (3)	0.071 (3)	0.002 (2)	0.020 (2)	0.001 (2)
C303	0.058 (3)	0.037 (3)	0.069 (3)	0.002 (2)	0.014 (2)	0.004 (2)
C3	0.057	0.042	0.082	−0.003	0.002	0.012
C304	0.048 (3)	0.041 (3)	0.041 (2)	−0.003 (2)	0.006 (2)	0.0057 (19)
C305	0.041 (3)	0.054 (3)	0.055 (3)	−0.002 (2)	0.006 (2)	0.000 (2)
C306	0.053 (3)	0.037 (3)	0.062 (3)	0.001 (2)	0.008 (2)	−0.001 (2)
C311	0.055 (3)	0.049 (3)	0.063 (3)	−0.009 (2)	0.023 (2)	−0.001 (2)
C312	0.051 (3)	0.044 (3)	0.100 (4)	−0.001 (2)	0.005 (3)	0.003 (3)
C313	0.052 (3)	0.036 (3)	0.076 (3)	−0.008 (2)	0.025 (3)	−0.011 (2)
C314	0.047 (3)	0.033 (3)	0.062 (3)	−0.006 (2)	0.024 (2)	−0.001 (2)
C315	0.060 (3)	0.045 (3)	0.071 (3)	0.005 (2)	0.023 (3)	0.001 (2)
C316	0.074 (4)	0.066 (3)	0.070 (3)	0.012 (3)	0.012 (3)	0.009 (3)
C317	0.091 (4)	0.071 (4)	0.064 (3)	−0.012 (3)	0.024 (3)	−0.008 (3)
C318	0.079 (4)	0.060 (3)	0.080 (4)	−0.014 (3)	0.041 (3)	−0.023 (3)
N11	0.047 (2)	0.033 (2)	0.048 (2)	0.0055 (16)	0.0018 (17)	−0.0006 (17)
N112	0.055 (2)	0.0287 (19)	0.049 (2)	−0.0004 (17)	0.0100 (18)	−0.0012 (16)
N211	0.052 (2)	0.046 (2)	0.064 (2)	0.0024 (17)	0.0133 (19)	−0.0065 (19)
N212	0.045 (2)	0.035 (2)	0.043 (2)	−0.0034 (16)	0.0112 (17)	−0.0002 (16)
N311	0.055 (3)	0.053 (2)	0.102 (3)	0.003 (2)	0.020 (2)	−0.022 (2)
N312	0.050 (2)	0.031 (2)	0.070 (3)	0.0010 (17)	0.021 (2)	−0.0033 (18)
O1	0.086 (2)	0.0419 (18)	0.111 (2)	−0.0004 (16)	0.0053 (18)	−0.0037 (16)
O2	0.067 (2)	0.065 (2)	0.098 (2)	0.0129 (16)	0.0141 (18)	−0.0146 (16)
O3	0.0550 (18)	0.0341 (16)	0.0788 (19)	0.0011 (13)	0.0234 (15)	−0.0074 (13)
O4	0.0542 (19)	0.0532 (18)	0.087 (2)	0.0013 (15)	0.0329 (17)	−0.0045 (15)
O5	0.094	0.096	0.095	−0.037	0.032	−0.014
N12	0.054	0.044	0.068	0.000	0.014	0.011
O7	0.064 (2)	0.0546 (19)	0.157 (3)	−0.0076 (16)	0.0104 (19)	0.0410 (18)
N111	0.058	0.045	0.070	0.009	0.022	0.008
O8	0.065 (2)	0.066 (2)	0.148 (3)	−0.0181 (17)	−0.011 (2)	0.0130 (18)

### Geometric parameters (Å, °)

**Table:**

C1—C6	1.365 (4)	C141—O3	1.324 (4)
C1—C2	1.376 (4)	C201—C202	1.367 (5)
C1—C11	1.521 (4)	C201—C206	1.369 (4)
C2—C3	1.397 (4)	C201—C211	1.510 (4)
C2—H1	0.9300	C202—C203	1.400 (4)
C5—C4	1.364 (4)	C202—H25	0.9300
C5—C6	1.383 (4)	C4—C3	1.361 (5)
C5—H3	0.9300	C4—C41	1.526 (5)
C6—H4	0.9300	C203—H26	0.9300
C341—O8	1.202 (4)	C206—C205	1.373 (5)
C341—O7	1.287 (4)	C206—H28	0.9300
C341—C304	1.491 (5)	C205—H27	0.9300
C11—N11	1.465 (4)	C211—N212	1.458 (3)
C11—H6	0.9700	C211—H30	0.9700
C11—H5	0.9700	C211—H29	0.9700
O6—C241	1.237 (4)	C212—N211	1.302 (4)
C12—N12	1.314 (4)	C212—N212	1.355 (4)
C12—N11	1.349 (4)	C212—H31	0.9300
C12—H7	0.9300	C213—N211	1.387 (4)
C13—N12	1.382 (4)	C213—C218	1.388 (4)
C13—C14	1.391 (4)	C213—C214	1.391 (4)
C13—C18	1.400 (4)	C214—C215	1.379 (4)
C14—C15	1.374 (4)	C214—N212	1.393 (4)
C14—N11	1.385 (4)	C215—C216	1.370 (4)
C15—C16	1.372 (4)	C215—H32	0.9300
C15—H8	0.9300	C216—C217	1.394 (5)
C16—C17	1.379 (5)	C216—H33	0.9300
C16—H9	0.9300	C41—O2	1.226 (4)
C17—C18	1.368 (5)	C41—O1	1.278 (5)
C17—H10	0.9300	C217—C218	1.376 (4)
C18—H11	0.9300	C217—H34	0.9300
C204—C205	1.355 (5)	C218—H35	0.9300
C204—C203	1.371 (4)	C301—C302	1.378 (4)
C204—C241	1.520 (5)	C301—C306	1.383 (4)
C101—C102	1.369 (4)	C301—C311	1.514 (4)
C101—C106	1.388 (4)	C302—C303	1.388 (4)
C101—C111	1.523 (4)	C302—H37	0.9300
C102—C103	1.390 (4)	C303—C304	1.377 (4)
C102—H13	0.9300	C303—H38	0.9300
C103—C104	1.387 (4)	C3—H2	0.9300
C103—H14	0.9300	C304—C305	1.377 (4)
C104—C105	1.370 (4)	C305—C306	1.379 (4)
C104—C141	1.488 (4)	C305—H39	0.9300
C105—C106	1.380 (4)	C306—H40	0.9300
C105—H15	0.9300	C311—N312	1.465 (4)
C106—H16	0.9300	C311—H41	0.9700
C111—N112	1.463 (4)	C311—H42	0.9700
C111—H17	0.9700	C312—N311	1.307 (4)
C111—H18	0.9700	C312—N312	1.353 (4)
C112—N111	1.310 (4)	C312—H43	0.9300
C112—N112	1.347 (4)	C313—C318	1.375 (5)
C112—H19	0.9300	C313—N311	1.383 (4)
C113—C118	1.383 (4)	C313—C314	1.402 (5)
C113—C114	1.390 (4)	C314—N312	1.377 (4)
C113—N111	1.391 (4)	C314—C315	1.380 (4)
C114—C115	1.378 (4)	C315—C316	1.381 (4)
C114—N112	1.388 (4)	C315—H44	0.9300
C115—C116	1.378 (4)	C316—C317	1.390 (5)
C115—H20	0.9300	C316—H45	0.9300
C116—C117	1.376 (5)	C317—C318	1.364 (5)
C116—H21	0.9300	C317—H46	0.9300
C117—C118	1.376 (5)	C318—H47	0.9300
C117—H22	0.9300	O1—H12	0.8200
C241—O5	1.225 (5)	O3—H24	0.8200
C118—H23	0.9300	O5—H36	0.8200
C141—O4	1.205 (4)	O7—H48	0.8200
			
C6—C1—C2	119.1 (4)	C204—C203—H26	120.3
C6—C1—C11	120.5 (4)	C202—C203—H26	120.3
C2—C1—C11	120.4 (4)	C201—C206—C205	119.6 (4)
C1—C2—C3	119.7 (4)	C201—C206—H28	120.2
C1—C2—H1	120.2	C205—C206—H28	120.2
C3—C2—H1	120.2	C204—C205—C206	122.9 (4)
C4—C5—C6	120.6 (4)	C204—C205—H27	118.5
C4—C5—H3	119.7	C206—C205—H27	118.5
C6—C5—H3	119.7	N212—C211—C201	111.7 (3)
C1—C6—C5	120.7 (4)	N212—C211—H30	109.3
C1—C6—H4	119.6	C201—C211—H30	109.3
C5—C6—H4	119.6	N212—C211—H29	109.3
O8—C341—O7	123.7 (4)	C201—C211—H29	109.3
O8—C341—C304	122.8 (4)	H30—C211—H29	107.9
O7—C341—C304	113.6 (4)	N211—C212—N212	114.4 (3)
N11—C11—C1	111.5 (3)	N211—C212—H31	122.8
N11—C11—H6	109.3	N212—C212—H31	122.8
C1—C11—H6	109.3	N211—C213—C218	129.6 (4)
N11—C11—H5	109.3	N211—C213—C214	110.8 (3)
C1—C11—H5	109.3	C218—C213—C214	119.6 (4)
H6—C11—H5	108.0	C215—C214—C213	123.8 (4)
N12—C12—N11	114.4 (3)	C215—C214—N212	131.6 (4)
N12—C12—H7	122.8	C213—C214—N212	104.5 (3)
N11—C12—H7	122.8	C216—C215—C214	115.6 (4)
N12—C13—C14	110.5 (3)	C216—C215—H32	122.2
N12—C13—C18	130.1 (4)	C214—C215—H32	122.2
C14—C13—C18	119.4 (4)	C215—C216—C217	121.8 (4)
C15—C14—N11	131.6 (4)	C215—C216—H33	119.1
C15—C14—C13	123.1 (4)	C217—C216—H33	119.1
N11—C14—C13	105.2 (3)	O2—C41—O1	126.7 (4)
C16—C15—C14	116.3 (4)	O2—C41—C4	119.6 (4)
C16—C15—H8	121.9	O1—C41—C4	113.6 (4)
C14—C15—H8	121.9	C218—C217—C216	122.0 (4)
C15—C16—C17	121.8 (4)	C218—C217—H34	119.0
C15—C16—H9	119.1	C216—C217—H34	119.0
C17—C16—H9	119.1	C217—C218—C213	117.1 (4)
C18—C17—C16	122.2 (4)	C217—C218—H35	121.5
C18—C17—H10	118.9	C213—C218—H35	121.5
C16—C17—H10	118.9	C302—C301—C306	118.6 (3)
C17—C18—C13	117.1 (4)	C302—C301—C311	121.3 (4)
C17—C18—H11	121.4	C306—C301—C311	120.1 (3)
C13—C18—H11	121.4	C301—C302—C303	120.2 (4)
C205—C204—C203	118.2 (4)	C301—C302—H37	119.9
C205—C204—C241	119.0 (4)	C303—C302—H37	119.9
C203—C204—C241	122.6 (4)	C304—C303—C302	120.8 (4)
C102—C101—C106	118.6 (4)	C304—C303—H38	119.6
C102—C101—C111	120.7 (3)	C302—C303—H38	119.6
C106—C101—C111	120.7 (4)	C4—C3—C2	120.7 (4)
C101—C102—C103	121.2 (4)	C4—C3—H2	119.7
C101—C102—H13	119.4	C2—C3—H2	119.7
C103—C102—H13	119.4	C303—C304—C305	119.2 (3)
C104—C103—C102	119.9 (4)	C303—C304—C341	122.0 (4)
C104—C103—H14	120.1	C305—C304—C341	118.8 (4)
C102—C103—H14	120.1	C304—C305—C306	120.0 (4)
C105—C104—C103	118.8 (3)	C304—C305—H39	120.0
C105—C104—C141	123.7 (3)	C306—C305—H39	120.0
C103—C104—C141	117.4 (4)	C305—C306—C301	121.2 (4)
C104—C105—C106	121.1 (4)	C305—C306—H40	119.4
C104—C105—H15	119.4	C301—C306—H40	119.4
C106—C105—H15	119.4	N312—C311—C301	111.9 (3)
C105—C106—C101	120.3 (4)	N312—C311—H41	109.2
C105—C106—H16	119.8	C301—C311—H41	109.2
C101—C106—H16	119.8	N312—C311—H42	109.2
N112—C111—C101	110.5 (3)	C301—C311—H42	109.2
N112—C111—H17	109.5	H41—C311—H42	107.9
C101—C111—H17	109.5	N311—C312—N312	113.6 (4)
N112—C111—H18	109.5	N311—C312—H43	123.2
C101—C111—H18	109.5	N312—C312—H43	123.2
H17—C111—H18	108.1	C318—C313—N311	130.0 (4)
N111—C112—N112	114.4 (3)	C318—C313—C314	120.6 (4)
N111—C112—H19	122.8	N311—C313—C314	109.3 (4)
N112—C112—H19	122.8	N312—C314—C315	132.5 (4)
C118—C113—C114	119.9 (4)	N312—C314—C313	105.5 (4)
C118—C113—N111	130.1 (4)	C315—C314—C313	121.9 (4)
C114—C113—N111	110.0 (3)	C314—C315—C316	116.3 (4)
C115—C114—N112	131.7 (4)	C314—C315—H44	121.9
C115—C114—C113	122.9 (4)	C316—C315—H44	121.9
N112—C114—C113	105.4 (3)	C315—C316—C317	121.8 (4)
C114—C115—C116	115.8 (4)	C315—C316—H45	119.1
C114—C115—H20	122.1	C317—C316—H45	119.1
C116—C115—H20	122.1	C318—C317—C316	121.6 (4)
C117—C116—C115	122.3 (4)	C318—C317—H46	119.2
C117—C116—H21	118.8	C316—C317—H46	119.2
C115—C116—H21	118.8	C317—C318—C313	117.8 (4)
C118—C117—C116	121.3 (4)	C317—C318—H47	121.1
C118—C117—H22	119.3	C313—C318—H47	121.1
C116—C117—H22	119.3	C12—N11—C14	106.0 (3)
O5—C241—O6	127.4 (4)	C12—N11—C11	127.5 (3)
O5—C241—C204	114.2 (4)	C14—N11—C11	126.3 (3)
O6—C241—C204	117.7 (4)	C112—N112—C114	106.1 (3)
C117—C118—C113	117.7 (4)	C112—N112—C111	127.3 (3)
C117—C118—H23	121.1	C114—N112—C111	126.5 (3)
C113—C118—H23	121.1	C212—N211—C213	104.1 (3)
O4—C141—O3	123.7 (4)	C212—N212—C214	106.2 (3)
O4—C141—C104	123.6 (4)	C212—N212—C211	127.4 (3)
O3—C141—C104	112.7 (4)	C214—N212—C211	126.3 (3)
C202—C201—C206	118.5 (4)	C312—N311—C313	105.1 (4)
C202—C201—C211	123.2 (4)	C312—N312—C314	106.5 (3)
C206—C201—C211	118.3 (4)	C312—N312—C311	125.9 (4)
C201—C202—C203	121.4 (4)	C314—N312—C311	127.6 (3)
C201—C202—H25	119.3	C41—O1—H12	109.5
C203—C202—H25	119.3	C141—O3—H24	109.5
C3—C4—C5	119.2 (4)	C241—O5—H36	109.5
C3—C4—C41	121.2 (4)	C12—N12—C13	103.9 (3)
C5—C4—C41	119.6 (4)	C341—O7—H48	109.5
C204—C203—C202	119.4 (4)	C112—N111—C113	104.1 (3)
			
C6—C1—C2—C3	−2.5 (5)	N211—C213—C218—C217	178.7 (4)
C11—C1—C2—C3	176.8 (3)	C214—C213—C218—C217	−1.2 (5)
C2—C1—C6—C5	2.5 (5)	C306—C301—C302—C303	−1.6 (5)
C11—C1—C6—C5	−176.8 (3)	C311—C301—C302—C303	177.6 (3)
C4—C5—C6—C1	0.5 (5)	C301—C302—C303—C304	1.5 (6)
C6—C1—C11—N11	59.7 (4)	C5—C4—C3—C2	3.3 (6)
C2—C1—C11—N11	−119.5 (4)	C41—C4—C3—C2	−175.1 (3)
N12—C13—C14—C15	179.6 (3)	C1—C2—C3—C4	−0.4 (6)
C18—C13—C14—C15	−0.7 (6)	C302—C303—C304—C305	−0.2 (6)
N12—C13—C14—N11	0.1 (4)	C302—C303—C304—C341	−176.4 (3)
C18—C13—C14—N11	179.8 (3)	O8—C341—C304—C303	−168.5 (4)
N11—C14—C15—C16	−179.9 (4)	O7—C341—C304—C303	11.1 (6)
C13—C14—C15—C16	0.7 (6)	O8—C341—C304—C305	15.3 (6)
C14—C15—C16—C17	−0.5 (6)	O7—C341—C304—C305	−165.2 (3)
C15—C16—C17—C18	0.3 (7)	C303—C304—C305—C306	−0.9 (5)
C16—C17—C18—C13	−0.3 (6)	C341—C304—C305—C306	175.4 (3)
N12—C13—C18—C17	−179.9 (4)	C304—C305—C306—C301	0.8 (6)
C14—C13—C18—C17	0.5 (5)	C302—C301—C306—C305	0.5 (6)
C106—C101—C102—C103	−2.6 (5)	C311—C301—C306—C305	−178.8 (3)
C111—C101—C102—C103	175.3 (3)	C302—C301—C311—N312	−110.0 (4)
C101—C102—C103—C104	−0.2 (5)	C306—C301—C311—N312	69.2 (4)
C102—C103—C104—C105	2.5 (5)	C318—C313—C314—N312	−177.1 (3)
C102—C103—C104—C141	−175.0 (3)	N311—C313—C314—N312	0.0 (4)
C103—C104—C105—C106	−1.8 (5)	C318—C313—C314—C315	0.7 (6)
C141—C104—C105—C106	175.4 (3)	N311—C313—C314—C315	177.8 (3)
C104—C105—C106—C101	−1.0 (5)	N312—C314—C315—C316	177.2 (4)
C102—C101—C106—C105	3.3 (5)	C313—C314—C315—C316	0.1 (6)
C111—C101—C106—C105	−174.7 (3)	C314—C315—C316—C317	−0.6 (6)
C102—C101—C111—N112	−66.8 (4)	C315—C316—C317—C318	0.4 (7)
C106—C101—C111—N112	111.1 (4)	C316—C317—C318—C313	0.4 (7)
C118—C113—C114—C115	1.5 (6)	N311—C313—C318—C317	−177.4 (4)
N111—C113—C114—C115	−177.6 (3)	C314—C313—C318—C317	−0.9 (6)
C118—C113—C114—N112	179.5 (3)	N12—C12—N11—C14	−0.8 (4)
N111—C113—C114—N112	0.4 (4)	N12—C12—N11—C11	174.6 (3)
N112—C114—C115—C116	−178.4 (4)	C15—C14—N11—C12	−179.0 (4)
C113—C114—C115—C116	−0.9 (6)	C13—C14—N11—C12	0.4 (4)
C114—C115—C116—C117	0.3 (6)	C15—C14—N11—C11	5.5 (6)
C115—C116—C117—C118	−0.1 (7)	C13—C14—N11—C11	−175.1 (3)
C205—C204—C241—O5	−163.5 (4)	C1—C11—N11—C12	−111.0 (4)
C203—C204—C241—O5	10.2 (6)	C1—C11—N11—C14	63.5 (4)
C205—C204—C241—O6	25.9 (6)	N111—C112—N112—C114	0.4 (4)
C203—C204—C241—O6	−160.4 (4)	N111—C112—N112—C111	−175.6 (3)
C116—C117—C118—C113	0.5 (7)	C115—C114—N112—C112	177.3 (4)
C114—C113—C118—C117	−1.2 (6)	C113—C114—N112—C112	−0.4 (4)
N111—C113—C118—C117	177.7 (4)	C115—C114—N112—C111	−6.7 (6)
C105—C104—C141—O4	178.3 (4)	C113—C114—N112—C111	175.6 (3)
C103—C104—C141—O4	−4.3 (5)	C101—C111—N112—C112	103.3 (4)
C105—C104—C141—O3	−2.8 (5)	C101—C111—N112—C114	−71.9 (4)
C103—C104—C141—O3	174.5 (3)	N212—C212—N211—C213	0.1 (4)
C206—C201—C202—C203	−1.8 (5)	C218—C213—N211—C212	−180.0 (4)
C211—C201—C202—C203	179.8 (3)	C214—C213—N211—C212	−0.1 (4)
C6—C5—C4—C3	−3.3 (5)	N211—C212—N212—C214	−0.1 (4)
C6—C5—C4—C41	175.1 (3)	N211—C212—N212—C211	−176.6 (3)
C205—C204—C203—C202	−1.6 (5)	C215—C214—N212—C212	178.0 (4)
C241—C204—C203—C202	−175.4 (3)	C213—C214—N212—C212	0.1 (4)
C201—C202—C203—C204	2.5 (5)	C215—C214—N212—C211	−5.5 (6)
C202—C201—C206—C205	0.2 (6)	C213—C214—N212—C211	176.6 (3)
C211—C201—C206—C205	178.7 (3)	C201—C211—N212—C212	107.1 (4)
C203—C204—C205—C206	0.1 (6)	C201—C211—N212—C214	−68.8 (4)
C241—C204—C205—C206	174.1 (4)	N312—C312—N311—C313	0.6 (5)
C201—C206—C205—C204	0.6 (6)	C318—C313—N311—C312	176.4 (4)
C202—C201—C211—N212	−49.9 (5)	C314—C313—N311—C312	−0.4 (5)
C206—C201—C211—N212	131.6 (3)	N311—C312—N312—C314	−0.6 (5)
N211—C213—C214—C215	−178.1 (3)	N311—C312—N312—C311	177.1 (3)
C218—C213—C214—C215	1.8 (6)	C315—C314—N312—C312	−177.2 (4)
N211—C213—C214—N212	0.0 (4)	C313—C314—N312—C312	0.3 (4)
C218—C213—C214—N212	179.9 (3)	C315—C314—N312—C311	5.2 (6)
C213—C214—C215—C216	−1.5 (5)	C313—C314—N312—C311	−177.3 (3)
N212—C214—C215—C216	−179.0 (3)	C301—C311—N312—C312	−87.8 (4)
C214—C215—C216—C217	0.6 (6)	C301—C311—N312—C314	89.3 (4)
C3—C4—C41—O2	−176.0 (4)	N11—C12—N12—C13	0.9 (4)
C5—C4—C41—O2	5.6 (6)	C14—C13—N12—C12	−0.6 (4)
C3—C4—C41—O1	2.9 (5)	C18—C13—N12—C12	179.8 (4)
C5—C4—C41—O1	−175.5 (3)	N112—C112—N111—C113	−0.2 (4)
C215—C216—C217—C218	−0.2 (7)	C118—C113—N111—C112	−179.1 (4)
C216—C217—C218—C213	0.5 (6)	C114—C113—N111—C112	−0.1 (4)

### Hydrogen-bond geometry (Å, °)

**Table:**

*D*—H···*A*	*D*—H	H···*A*	*D*···*A*	*D*—H···*A*
O1—H12···N12^i^	0.82	1.92	2.693 (4)	157.
O3—H24···N111^ii^	0.82	1.85	2.613 (4)	154.
O5—H36···N211^ii^	0.82	1.92	2.711 (4)	162.
O7—H48···N311^iii^	0.82	1.84	2.628 (4)	160.

Symmetry codes: (i) −*x*+2, *y*−1/2, −*z*+1/2; (ii) −*x*+1, *y*+1/2, −*z*+1/2; (iii) −*x*, *y*−1/2, −*z*+1/2.

## References

[bb1] Brandenburg, K. (2000). *DIAMOND* Crystal Impact GbR, Bonn, Germany.

[bb2] Bruker (2008). *APEX2* and *SAINT* Bruker AXS Inc., Madison, wisconsin, USA.

[bb3] Das, M. C. & Bharadwaj, P. K. (2009). *J. Am. Chem. Soc.* **131**, 10942–10943. 10.1021/ja9006035 19621875

[bb4] Hua, Q., Zhao, Y., Xu, G.-C., Chen, M.-S., Su, Z., Cai, K. & Sun, W.-Y. (2010). *Cryst. Growth Des.* **10**, 2553–2562.

[bb5] Kuai, H.-W. & Cheng, X.-C. (2011*a*). *Acta Cryst.* E**67**, o2787. 10.1107/S1600536811039043 PMC3201311 22058821

[bb6] Kuai, H.-W. & Cheng, X.-C. (2011*b*). *Acta Cryst.* E**67**, o3014. 10.1107/S1600536811042838 PMC3247413 22220031

[bb7] Sheldrick, G. M. (1996). *SADABS* University of Göttingen, Germany.

[bb8] Sheldrick, G. M. (2008). *Acta Cryst.* A**64**, 112–122. 10.1107/S0108767307043930 18156677

